# Autochthonous Biological Resources for the Production of Regional Craft Beers: Exploring Possible Contributions of Cereals, Hops, Microbes, and Other Ingredients

**DOI:** 10.3390/foods10081831

**Published:** 2021-08-07

**Authors:** Nicola De Simone, Pasquale Russo, Maria Tufariello, Mariagiovanna Fragasso, Michele Solimando, Vittorio Capozzi, Francesco Grieco, Giuseppe Spano

**Affiliations:** 1 Department of Agriculture, Food, Natural Science, Engineering, University of Foggia, Via Napoli 25, 71122 Foggia, Italy; nicola.desimone@unifg.it (N.D.S.); pasquale.russo@unifg.it (P.R.); mariagiovanna.fragasso@gmail.com (M.F.); giuseppe.spano@unifg.it (G.S.); 2 Institute of Sciences of Food Production, National Research Council of Italy (CNR), Via Prov.le Lecce-Monteroni, 73100 Lecce, Italy; maria.tufariello@ispa.cnr.it (M.T.); francesco.grieco@ispa.cnr.it (F.G.); 3 Rebeers, Microbrewery, Viale degli Artigiani 30, 71121 Foggia, Italy; michele@rebeers.it; 4 Institute of Sciences of Food Production, National Research Council (CNR), c/o CS-DAT, Via Michele Protano, 71121 Foggia, Italy

**Keywords:** craft beer, ingredient, autochthonous, regionalisation, brewing, cereal, hop, yeasts, bacteria, biodiversity

## Abstract

Selected biological resources used as raw materials in beer production are important drivers of innovation and segmentation in the dynamic market of craft beers. Among these resources, local/regional ingredients have several benefits, such as strengthening the connection with territories, enhancing the added value of the final products, and reducing supply costs and environmental impacts. It is assumed that specific ingredients provide differences in flavours, aromas, and, more generally, sensory attributes of the final products. In particular, of interest are ingredients with features attributable and/or linked to a specific geographical origin. This review encompasses the potential contribution and exploitation of biodiversity in the main classes of beer inputs, such as cereals, hops, microbes, and adjuncts, with a specific emphasis on autochthonous biological resources, detailing the innovative paths already explored and documented in the scientific literature. This dissertation proposes an overview of the impact on beer quality for each raw material category, highlighting the benefits and limitations that influence its concrete applications and scale-up, from the field to the stain. The topics explored promote, in the sector of craft beers, trends already capitalised in the production of other alcoholic beverages, such as the preservation and revalorisation of minor and autochthonous varieties, the exploitation of yeast and bacteria strains isolated from specific sites/plant varieties, and the valorisation of the effects of peculiar terroirs on the quality of agricultural products. Finally, the examined tendencies contribute toward reducing the environmental impacts of craft beer manufacturing, and are in line with sustainable development of food systems, increasing the economic driver of biodiversity preservation.

## 1. Introduction

Beer is one of the most consumed and appreciated beverages worldwide. The global beer market is constantly growing, supported by the emerging segments of non-alcoholic (i.e., alcohol-free and low-alcohol beers), gluten-free, organic, and craft beers [1]. In particular, craft beers are produced by small and independent microbreweries, which have rapidly risen worldwide due to increased interest in artisanal products [2]. Nevertheless, several factors, such as the competitive market, energy costs, scale, and taxation, appear to undermine the economic sustainability of microbreweries. For these reasons, the diversification and extension of the range of products are becoming increasingly important, leading to interest in finding new beer styles and improving the added value of marketed beer.

**Figure 1:**
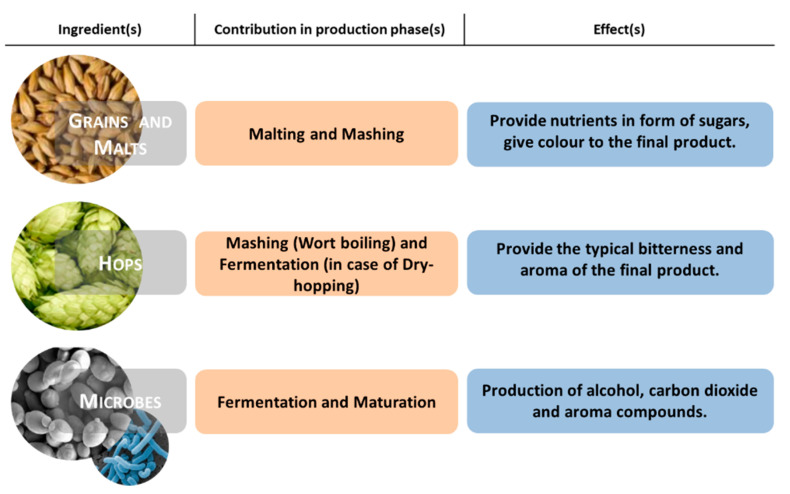
Contribution of the main ingredients in the beer production process and their effects on the final product.

Considering the above, there is increased interest in the ingredients and raw materials involved in the beer production process, which could be simplified into four steps: malting, mashing, fermentation, and maturation. The principal ingredients are mainly water, barley malt, hops, and yeast. Figure 1 shows the participation of the production phases of the main raw materials and their effects on the final product. In addition, the list of traditional ingredients can be extended according to the beer style and the breweries. In fact, it is assumed that specific raw materials could provide different flavours, aromas, and sensory attributes [3]. In this regard, the use of autochthonous biological inputs could provide several benefits, e.g., reducing the production and supply costs and creating a strong connection with territories that give added value to the final products.

Water (including mineral water) is also used in the mashing stage, and is often crucial in connecting with a given site. Water chemical parameters influence several parameters, such as wort pH, yeast flocculation, α-amylase activity, hop utilisation, colour, mouthfeel, and palatability of the final product [3,4,5,6]. Therefore, the water used at famous brewing cities, e.g., in Dublin, Dortmund, Vienna, Munich, London, and Edinburgh, are utilized as references for the respective beer styles [7]. However, mineral composition, and the concentration of certain ions, such as carbonate, sulphate, iron, and manganese, are often adjusted to avoid unfavourable sensory attributes, colours, and tastes [8], and to comply with regional guidelines (such as in Germany) [8], or to suit the parameters given by regions/cities in order to obtain the same beer styles [4].

The brewing features of autochthonous biological resources, such as cereals, malts, hops, microbes, and adjuncts of biological origin, are differently influenced by specific soil features and environmental factors [9,10], demonstrating that regional attributes may also be promoted differently in the beer production chain. This association—known as *terroir* —was already described for other products related to specific regions, having particular interest in the wine industry, and could take advantage of the production of regional craft beers [11].

This review aims to provide an overview of the raw materials of beer production, focusing on autochthonous biological resources used as raw material associated with a given geographical context. The proposed analysis comprehends the advantages in exploiting the regional biodiversity to design and improve the unique content of regional craft beers.

## 2. Ingredients

### 2.1. Grains and Malted Cereals

Cereals are the main ingredients for beer production; they provide nutritional sources for the fermentation progress, principally in the form of sugars. The main carbohydrate in the grain is starch, which is unfermentable and unusable by brewing yeasts. For this reason, prior to being used in the brewing process, cereals must be malted to promote the synthesis of the enzymes required for starch hydrolysis in fermentable sugars. After the germination, malts are dried and kilned. At this stage, different kilning procedures (time and temperature) allow obtaining different malt types, such as base malts (pale), speciality (caramel), and roasted (chocolate, coffee) [12]. Moreover, a wide range of malts, covering a plethora of beer styles, is available on the market.

Barley (*Hordeum vulgare* L.) and wheat (*Triticum durum* Desf. and *Triticum aestivum* L.) are the main cereals used in the malt industries. Among these, barley malt accounts for about 90% of worldwide beer production [12]. Whereas wheat malt is used in proportion with barley for the production of wheat (or sometimes ‘weiss’ or ‘weizen’) beers, characterised by distinctive cloudiness and persistent foam due to the major protein content of wheat malt [13]. Belgium and Northern Germany are historically known for the production of traditional beers brewed with raw (Belgian Witbier) and malted (German Weißbier) wheat, which are included in the list of classical beer styles, and are now produced and exported worldwide [13].

The use of autochthonous cultivars of cereals is one of the most explored strategies for the regionalisation of beer production. In this regard, cereals used in brewing vary among countries and as a function of the desired features of the final products (Table 1). Moreover, some of these cereals could be useful in producing beer with particular added value (e.g., gluten-free beer [14]), providing different nutritional and sensory properties [15], and reducing production costs [16]. Nevertheless, integrating the malting stage is considered a ‘bottleneck’ in the regionalisation of the beer industry [17], and autochthonous cereals are often used raw and unmalted as partial substitutes for barley malt.

The Mediterranean area is characterised by the presence of several cereal cultivars, in some cases only locally diffused, which could be exploited to improve segmentation in brewing regionalisation. Ancient wheat varieties have recently gained popularity due to their nutraceutical properties, from their higher concentrations of flavonoids, fibre, and minerals [41,42,43]. In Sardinia, Italy, a craft beer brewed with the old Italian wheat cultivar ‘Senatore Cappelli‘ as an unmalted supplement (40%) was compared with two industrial wheat beers, resulting in higher polyphenol content and more balanced taste [18]. Other *Triticum* spp., such as einkorn (*Triticum monococcum* L.), emmer (*Triticum dicoccum* L.) and spelt (*Triticum spelta* L.), are used to obtain malts with higher antioxidant activities and total polyphenol content [20], characterised by the presence of more fibre, lower gluten content [19], and low extract yields [21]. New hybrids were recently assessed for malt and beer production. Among those, Tritordeum (x *Tritordeum martinii*), obtained by crossing wild barley with wheat, was comparable to barley in saccharification time, lautering, as well as colour and turbidity, but a slightly acidifying effect and higher free amino nitrogen were observed [22]. Whereas, Triticale (x *Triticosecale*), a hybrid of durum wheat and rye, is suitable for malt production because of its high extraction capacity, high diastatic power, and short saccharification time, but with higher acidity and lower esters and isoamyl alcohol content [23].

Oat (*Avena sativa* L.) is considered a functional cereal due to its pronounced level of antioxidants, fibre, and β-glucan. Compared to barley, oat is characterised by a higher husk content, which improves lautering performance, but leads to lower extract content, malted or unmalted [15,29]. Beer produced entirely from oat malts also has higher pH, lower alcohol content, and an intense berry flavour [29], while any significant difference can be found in the protein profile and in the fermentation trend [29]. The enhancement of extract and alcohol content by using exogenous enzymes, such as β-glucanases, amylases, neutral proteases, and hemicellulases, was reported on in the literature [15].

Rice (*Oryza sativa* L.), due to its high starch content, is sometimes used in brewing as a supplement to increase the sugar content of the mash [24,25]. Different trials were carried out in the production of rice beers [24,25,26,27,28]. Nevertheless, its protein and enzymatic profiles are lower than barley, and, furthermore, its starch does not entirely break up during the malting and mashing stage [44]. Despite the flat sensory profile and soft pale colour being reported in the first trials [24,25,26], optimisation of rice malt production resulted in an enhancement of flavour, taste, colour, and body of all rice beers, with encouraging results recently obtained [27,28].

Other grains, such as corn and sorghum, although they are considered as supplements for beer production, are commonly used to ferment traditional beers in South America and Africa, respectively, where they are the most cultivated cereals. Corn (*Zea mays*) is used to produce several indigenous South- and Mesoamerican beers, commonly known as Chicha [45]. Among these, *Chicha de Guiñapo*, traditional of Arequipa (Peru), is based on malted-pigmented corn [46]. Recently, pigmented corn malt was proposed as the main ingredient in different beer-style productions [31,32]. Whereas sorghum-based African beers are characterised by a slight alcohol content (3.6% ethanol), brown colour, and acid pH (4.15 on average) due to the alcoholic and lactic co-fermentation [33]. However, some problems, such as incomplete saccharification, residue of insoluble materials, increased viscosity, and low free amino nitrogen content, occur in brewing with sorghum malt [34]. For these reasons, it is necessary to develop an appropriate process to improve malting and mashing conditions and the final sensory aspects. In this regard, the use of different formulations of exogenous enzymes has been evaluated. For example, β-amylase increases the amount of fermentable sugars [35], whereas amyloglucosidase treatment improves wort yield, resulting in higher alcohol content [35,36]. Alternatively, the α-amylase, β-amylase, and amyloglucosidase activities of sorghum malts could be enhanced by the addition of koji (*Aspergillus oryzae*) [37].

### 2.2. Hops

Hops are the unfertilised female inflorescence of the perennial climbing vine *Humulus lupulus* L., belonging to the family Cannabaceae. The importance of hop is related to its bitterness, which contributes to the characteristic aroma and flavour of beer. Hops also enhance foam formation and stability and have antibacterial properties protecting against spoilage by certain microorganisms [47]. Their key compounds for brewing are resins and essential oils. Among the resin fraction, iso-α-acids, which originate when hops are added during wort boiling, are the most significant bittering compounds. Essential oils, mainly terpenoids, are extracted during late- and dry-hopping and are responsible for the beer aroma [48]. The perceived sensorial attributes depend on the hop varieties [49] and different commercial preparations (whole leaf, pellets, extracts) [3]. Brewers formerly divide hops into two groups based on their bittering or aroma contributions [50]. Among the most known cultivars, *Target, Admiral, Nugget, Pride of Ringwood,* and *Super Pride* release a high quantity of bittering compounds, while *Fuggles, Goldings, Saaz, Willamette, Cascade,* and *Cluster* varieties provide a pleasant hoppy aroma [3]. Hop cultivars have a strong connection with the geographical contest. In fact, they are also divided into groups, such as American, German, British, European, and others, according to their origin. For example, aroma hop varieties, such as *Hallertau* and *Hersbrucker,* are denominated according to a German region and city, respectively, and are among the most cultivated hops in Germany. *Saaz* is termed with the German name Žatec (Saaz), a city in the Czech Republic, and it is a traditional ingredient of Pilsner beer. Nevertheless, the United States and Germany are the most important producers, with about 50,000 tons for each, and Germany as the larger exporter, with 25,000 tons exported worldwide [51]. In this regard, there is growing interest in the definition of hop *terroir* and in presenting the differences among cultivars grown in different geographical contests. Table 2 shows the difference in terms of quality among hops from different *terroir*. The dual-purpose American hop *Amarillo* grown in Idaho has lower citrusy and floral notes but a more fruity, spicy, and resinous odour than Washington [52]. In the same regions, the effect of *terroir* was later confirmed for hexyl glucoside content, a green leaf volatile with a grassy aroma, in twenty-three hop cultivars [53]. Similarly, *Cascade* from the Hallertau has more polyphenols and esters, but lower terpene content than those grown in Yakima (Washington, DC, USA) [54]. On the contrary, the same variety cultivated in Sardinia (Italy) had essential oils and acid content comparable to those farmed in the US [55]. In this way, recent research compared the volatile fingerprint and the acid profile of 15 commercial international hop cultivars, grown in an experimental field of Central Italy, with their standard characteristics, discovering desirable acids and terpene content in the cultivars *Chinook*, *Yeoman,* and *Hallertau* [56].

The breeding of new varieties is another way to expand the plethora of hop cultivars, and to select those suitable for specific regions. In France, the variety *Strisselspalt* was used as a parental strain to obtain the new ones, *Bouclier* and *Triskel*, which showed significantly different terpenoid profiles [57]. While the variety *Kazbek* was bred in Czech Republic as the first aroma hops variety with a specific citrus-like aroma for Pilsner beer [58]. *Sorachi Ace*, the most known Japanese hop, has a significant amount of geranic acid, enhancing the varietal aroma of the hop-derived terpenoids [59].

Evaluating the brewing potential of wild hop varieties is one of the new tools used in the regionalisation of beer production. Mongelli et al. [60] characterised the aromatic profiles of 22 Italian wild hop genotypes. The low essential oils and bitter acidic content of some of these ecotypes suggest a potential exploitation, for a dual-purpose or for dry hopping hops. In addition, a comparison between wild hops collected from different regions of North America and the Caucasus showed significant differences in the contents of α- and β-acids, cohumulone, and colupulone amount [61].

### 2.3. Microbes

Among microbes, yeast plays the most important role in beer production, as it ferments the wort, metabolises the sugar, and produces the compounds that define the peculiar characteristics of the beverage. It is also crucial for defining the flavour and aroma of the final products, including the synthesis of higher alcohols, esters, aldehydes, and organic acids [63], but also the bioconversion of hop-derived compounds [64,65,66]. For this reason, yeast starter cultures, in some cases, are protected and included in the list of autochthonous and/or traditional ingredients of regional beers (e.g., Münchener Bier) [67]. In brewing, yeasts are formerly divided into two major groups, based on beer-style, working temperatures, and flocculation ability. In the first group, there are those belonging to the species *Saccharomyces cerevisiae*, which are suitable for ale beers, capable of fermenting at warmer temperatures (16–22 °C) and able to flocculate or aggregate at the top of the vessel once fermentation is complete. In the second group, there are natural hybrids between *S. cerevisiae* and *S. bayanus* species, known as *S. pastorianus* (syn. *S. carlsbergensis*), suitable for lager-style; they ferment at lower temperatures (6–16 °C) and settle to the bottom of the vessel at the end of fermentation [68]. The two styles also need different periods of maturation: lager beers undergo a long, low-temperature period of ageing (known as lagering), while ale beers are usually mature in a short period [68]. The world beer production is represented by 90% for lager, and 5% for ale, while the other 5% is produced by spontaneous fermentation [68]. The latter has a particularly sour taste resulting from the sequential or contemporary fermentation by different microbes, among which, *Saccharomyces*, *Dekkera*/*Brettanomyces,* and lactic acid bacteria (LAB), such as *Lactobacillus* and *Pediococcus*, are considered the most important for the final beer character [69]. In the traditional Belgian lambic style, and its analogous American coolship ale, the wort is exposed to air during cooling and then transferred to wood barrels used in previous fermentations, resulting in a spontaneous inoculation by a consortium of microorganisms [69,70,71,72,73]. A microbial population of more than 2000 strains has been documented in lambic fermentation [69]. Nevertheless, the complete maturation of these kinds of beers can take many years, making it difficult to obtain a suitable quantity of products for commercial purposes [74].

The demand for novel starter cultures for brewing is increasing, and brewers and scientists are converging on the selection of those that could bring added value to the final products. Nowadays, the definition of microbial *terroir* has assumed relevant significance in wine production [75,76,77,78]; however, this approach has only recently gained popularity among breweries. In brewing, microbial *terroir* could be associated with the use of native microbes, isolated from traditional beer ingredients, but also with those strains isolated from other autochthonous biological resources. In fact, different research trends are focusing on improving the microbial biodiversity useful for beer production, including the exploration of the brewing potential of different groups of microorganisms, such as *Saccharomyces* strains isolated from other fermented food and beverage hybrids of the *Saccharomyces* genus and non-*Saccharomyces* species [79]. Thus, the features of relevant interest comprise sugar utilisation (mainly maltose and maltotriose), hops and ethanol tolerance, and ethanol and flavouring compound production (e.g., esters and higher alcohols) [80]. Table 3 reassumes the main microbial strains recently investigated for their impact on beer quality.

The *Saccharomyces* species are responsible for the primary fermentation of a large variety of fermented food and beverages. However, the ability to metabolise maltose and maltotriose is not widespread, thus restricting the brewing potential only to a few species [80]. Rossi and co-workers [101] compared the fermentative ability in laboratory-scale fermentation and volatile profiles of different *S. cerevisiae* strains isolated from grape must, bakery, and wine and apple stillage. The authors selected a baking yeast as the most promising strain, leading to features in line with ale profiles and with a pronounced contribution in esters (above threshold). In this contest, sourdough could represent an important source of biodiversity when selecting autochthonous strains suitable for craft beer production. This idea is supported by the fact that some regional beers, such as Finnish Sahti beers, recognised in the European Union as ‘Traditional Speciality Guaranteed’, are traditionally fermented using baking yeast strains [102]. In this light, Ripari et al. [103,104] reported for the first time the utilisation of the whole microbiota of artisanal sourdough, comprised of yeasts and LAB, to obtain sour beers inspired by lambic fermentation. Other studies confirmed the potential of sourdough yeasts. For example, in Sardinia (Italy), two *S. cerevisiae* strains from artisanal sourdoughs were selected to produce wheat beers from the Italian cultivars ‘Senatore Cappelli’ [82,83,84]. Among those, *S. cerevisiae* S-38 has shown physical–chemical, volatile, and sensory characteristics comparable to the commercial strain Safale S-33 (Fermentis, Lesaffre, Marcq-en-Baroeul, France) [83], whereas *S. cerevisiae* S-42 had higher ethanol content, lower pH, and a higher content of esters [82]. While Catallo et al. [85] used a sourdough strain as a parental strain in a de novo hybridisation to obtain a hybrid for lager beer fermentation. The authors reported that the hybrid inherited the ability to produce positive aroma compounds, such as 3-methylbutylacetate, ethyl acetate, and ethyl hexanoate by the sourdough strain, together with the efficient utilisation of maltotriose from the other parental strain [85].

Non-*Saccharomyces* yeasts have limited fermentation performance and are less tolerant to ethanol, but they produce volatile compounds, contributing to the sensory characteristics of several fermented beverages [105]. For this reason, different genera have recently been investigated for their contributions to brewing [106]. Among these, *Dekkera*/*Brettanomyces* strains are the most known as they are considered the main spoilers in beer and wine production, but if applied correctly, they contribute to the production of exotic flavours and the aroma complexity of speciality beers [107]. The enhancement of aromatic flavour in beer by different *Torulaspora delbrueckii* strains of oenological origins, to the production of isoamyl acetate, has been well documented, in both pure and mixed fermentations with commercial starters [92,93,94,108,109]. Meanwhile, the strains unable to ferment maltose *Saccharomycodes ludwigii* and *Zygosaccharomyces rouxii* have been studied to produce special beer styles, such as low-alcohol and alcohol-free beer [91]. Differently, the use of species able in producing lactic acid, such as *Lachancea thermotolerans* [89,110], *L. fermentati*, *Hanseniaspora vineae*, *Schizosaccharomyces japonicus*, and *Wickerhamomyces anomalus* [111] has been investigated for the production of sour beers in a single step of fermentation. Different methods for producing sour beers, such as kettle souring, sequenced, and mixed controlled fermentation, were recently explored [73,112]. Sour beers, such as the German Berliner Weisse and Gose, and the spontaneously fermented Belgian lambic and American Coolship Ale, have an intentional acidic taste given by the fermentation of acid-producing bacteria, mainly LAB, aside from brewing yeasts [112]. In addition, since LAB could be isolated from several food matrices, including brewing ingredients [97], they could contribute essentially to the regionalisation. In this regard, *L. amylovorus* FST2.11, isolated from the brewing environment, was tested with different acidification methods, suggesting kettle souring as the best practice to obtain sour beer with minimal organoleptic failures [97]. Contrarily, Dysvik et al. [95] showed that beer co-fermented with LAB strains, such as *L. plantarum* and *L. brevis* BSO464, had an increased intensity in fruity odour and higher total flavour intensity, respectively. These suggest that organoleptic attributes could be species- and/or strain-dependent, rather than method-dependent, since pre-acidification of the wort did not surpass that obtained by chemical acidification [113].

### 2.4. Adjuncts

The use of unusual ingredients is widespread among breweries, and although they are not essential for beer production—they are often used for the production of speciality beers. However, concerning the high number of commercially available products belonging to this category, only a few data are available in the literature. Among adjuncts, fruits and spices are often used to enrich the flavour complexity of certain beer styles. For example, orange peel and coriander are traditional ingredients of Belgian Witbier [114]. Fruits (as whole or as juices) are among the most studied supplements, and they are already present in many commercial products [115]. In Belgian lambic-style, for example, whole raspberries (*Rubus idaeus* L.) and tart cherries (*Prunus cerasus* L.) are traditionally used in the maturation of Framboise and Kriek beers, respectively [74]. Recently, the Beer Judge Certification Program recognised the Italian Grape Ale (IGA) as the first Italian style, which could be an expression of the regional biodiversity, promoted by the wide availability of grape cultivars [116]. In IGA, grapes can be added up to 40% during mashing, fermentation, and maturation. In addition, the use of several fruits, such as bananas or persimmon, has been reported in the literature. In both forms, fruit supplementation increases fermentable sugars [117,118], adds flavour and fruity aroma [117], modifies the colour due to the solubilisation of pigments, such as carotenoids [117] and anthocyanins [119,120], enhances antioxidant activity and total phenolics [115], increases bioactive compounds [115,121], adds a different degree of acidity [117,119], and increases alcohol content [118]. In addition, these supplementary ingredients could represent relevant links to specific regions (Table 4).

Other ingredients can also be added to contribute to bitterness. In fact, before hops introduction, beers were initially flavoured with a mix of herbs and spices called gruit, which contained several herbs and spices [50]. Therefore, other bittering plants were investigated as hops substitutes, including where hop cultivation was not suitable. For instance, artichoke and carqueja were positively evaluated in Brazil as total substitutes because of their good sensorial acceptance and the absence of negative effects on physicochemical characteristics [127,128].

The regionality of the adjuncts is only poorly evaluated; however, intriguingly, attention is placed on the use of by-products from the food industry [96,132], with the incremental aim of reducing food waste and its economic weight. 

## 3. Conclusions

The selection of biological resources used as inputs in beer production is an important process to achieve a desired segmentation and diversification in the competitive market of craft beer [133]. This review paper encompasses the potential exploitation of biodiversity belonging to the main classes of raw beer materials (i.e., grains and malted cereals, hops, microbes, and adjuncts), with a specific emphasis on autochthonous resources. In fact, regional ingredients can create strong connections with territories, attracting consumers and enhancing the added value of the final products [134,135,136]. Dynamics that favour the exploitation of trends already well explored in the wine sector, such as (i) the preservation and revalorisation of minor and autochthonous varieties; (ii) the exploitation of yeast and bacteria strains isolated from specific sites/varieties; and (iii) the valorisation of the effects of peculiar *terroirs* on the quality of agricultural products [137,138,139,140]. In addition, the use of autochthonous resources is well in line with the potential of “agribreweries” businesses—agrarian enterprises recognised by specific national legislation that have to use at least 51% of self-produced raw materials for their brewing processes [141].

This work proposed a detailed dissertation about the innovative paths already explored and documented in the scientific literature. Each ingredient has peculiar characteristics [133,142], and thus, benefits and limitations affecting its concrete applications and scale-up to brewery points. From the field to the stein—there are different weaknesses and strengths affecting future development. In regard to agronomic practices, one limiting factor to the ‘regionalisation’ of hop production is the extensive use of American and German cultivars because of their well-known properties. However, growing interest in the craft beer sector has already promoted local cultivation of these most diffused hop cultivars, suggesting the possible exploitation of a sort of ‘terroir’ effect [143]. On the other hand, as the use of wild hops is only at the beginning, as a function of their genotypic stabilities, new features could be exploited in different beer styles (from low hoppy to IPA). From a technical point of view, the possibility of performing the malting stage on a small scale is among the “bottleneck” in the revalorisation of minor and autochthonous cereal varieties in brewing [144]. Local unmalted grains are already used in several beer recipes of local microbreweries, and different pilot plants for micro malting are growing in different contexts, which will allow, in the near future, a growing plethora of opportunities to reach the market. Due to the huge number of possible new malted/unmalted integrations, one key point would be the development of balanced recipes to interpret the different beer segmentations and meet consumers’ needs. In regard to fermentation, the exploitation of different microbial resources has assumed relevance in recent years due to the diversity of final products that could be obtained. This is confirmed by the recent expansion of starter cultures, which, nowadays, comprise of not only *Saccharomyces* strains but also non-*Saccharomyces* and lactic acid bacteria [145,146], as is the case with commercial starter cultures for oenological production [147,148,149]. In this regard, a sector of craft beer could be a model sector of regionalisation by means of ‘cross-over’ microbial innovations [150]. In fact, craft brewing is often considered a new production chain, in regional contexts, and could take advantage of the wide microbial diversity isolated and characterised from regional traditional fermented foods and beverages [138,139]. Thus, the screening of existing microbial collections to assess to what extent they are reservoirs of potential brewing microbes is an emerging field that could improve the relationship of craft beer with specific geographical origins. Moreover, the development of complex microbial starters, comprised of different microbial strains in co- or sequential fermentation, could be one of the main challenges in beer investigations oriented to regionalisation and market segmentation [149].

Finally, the design of beers connected with specific geographic contexts, and the need of regional supply chains for cereals, hops, and microbial resources, could contribute to the reduction of the production and supply costs and the environmental impact of craft beer, in line with the sustainable development of food systems, increasing the economic driver of biodiversity preservation [17,133,151,152,153,154,155].

## Figures and Tables

**Table 1:** Cultivars, origins, and impact of grains that were recently investigated for their brewing potential.

Grains	Regions	Impact on Beer Quality	Ref.
Durum wheat (cv. Senatore Cappelli)	Sardinia (IT)	High polyphenol content, balanced taste, low sweetness.	[18]
Einkorn, emmer and spelt	Italy, Hungary	Higher antioxidant activity and polyphenol content, more fibre, lower gluten content, and low extract yields.	[19,20,21]
Tritordeum (cv. Bulel)	Spain	Addition of slight acidity and higher free amino nitrogen.	[22]
Triticale (cv. Remiko)	Poland	Higher acidity, lower esters and isoamyl alcohol content.	[23]
Rice (cv. Loto)	Italy	Acceptable alcoholic content (3.5–4.5% vol.), good foam stability, rather poor in body and mouthfeel.	[24]
Rice (cv. Centauro)	Italy	Pale colour, not persistent foam, flat sensory characteristics. Optimisation of malt production improves colour and flavour.	[25,26,27,28]
Oat (cv. Koneser)	Finland	Increased protein content in the wort and prolonged filtration time.	[15]
Oat (cv. Raisio)	Finland	High pH and low alcohol content, strong berry flavour and low amount of staling compounds during ageing.	[29]
Corn (cv. Nzaka-nzaka)	Congo	Poor foam stability, saccharification needs of exogenous α-amylase.	[30]
Pigmented Corn (cv. Chalqueño)	Mexico	Low-alcohol beer with polyphenols and anthocyanins with antioxidant properties.	[31,32]
Sorghum	African countries	Slight alcoholic (3.6%), brown colour, and acid pH (4.15 on average) due to the alcoholic and lactic co-fermentation. Incomplete saccharification, residue of insoluble materials, increased viscosity.	[33,34]
Red Sorghum (cv. DKS-74)	Mexico	Exogenous enzymes treatment yields glucose and alcohol content similar to barley-malt beer.	[34,35,36,37]
Quinoa	Bolivia	Higher foam stability, lower level of soluble nitrogen, and more than twice the amount of fat; positive effect on the overall sensorial quality.	[38]
Rye (cv. Dukato)	Belgium	Increased beer viscosity, higher palate-fullness.	[39]
Teff (cv. Witkop)	South Africa	Higher content of glucose and a lower content of maltose, higher sweetness, fruity aroma, with little body.	[40]

**Table 2:** Cultivars, origins, and impact of hops recently investigated for their brewing potential.

Hops’ Varieties	Region	Impact on Beer Quality	Ref.
Kazbek	Czech Republic	Low content of alpha acids, citrus-like aroma due to geranyl esters content of essential oil fraction.	[58]
Aramis	France	Terpenoid profile similar to the parental variety Strisselspalt, gives spicy and herbal notes to the beer.	[57]
Triskel	France	High concentration of monoterpenoids, especially linalool, which bring a floral note to beer.	[57]
Amarillo	Idaho (US)	Lower citrusy and floral notes, but higher fruity, spicy, and resinous odour descriptions.	[52]
Cascade	Hallertau (DE)	Higher content of polyphenols and esters, such as isobutyl-isobutyrate and 2-methylbutyl-2-methylpropanoate.	[54]
Cascade	Washington (US)	Higher linalool contents with respect to those grown in Hallertau (DE).	[54]
Cascade	Sardinia (IT)	Essential oil and the α-/β-acids in the same range of those cultivated in the US.	[55]
Cascade	Brazil	Higher content of farnesene and selinene, but lower levels of humulene and myrcene respect to the US grown crops.	[62]
Sorachi Ace	Hokkaido (JP)	It contains a unique volatile compound, geranic acid, which enhances the aroma contribution of terpenoids at sub-threshold levels.	[59]
Wild hops	Italy	Selinenes, α-acids, trans-β-farnesene, and α-caryophyllene/β-humulene ratio are the main contributors and have a higher content of xanthohumol and α-acids among European wild hops.	[60]
Wild hops	Canada	High content of myrcene and low contents of humulene, farnesene, and selinenes.	[61]
Wild hops	Caucasus	Significantly lower cohumulone content.	[61]

**Table 3:** Species, strains, origin, and impact of microbes recently investigated for their brewing potential.

	Species/Strains	Source	Region	Impact on the Beer Quality	Ref.
*Saccharomyces* spp.	*S. cerevisiae*	Wine	Italy	Higher fruity and flowery aroma compounds in bottle re-fermentation.	[81]
	*S. cerevisiae* S-42	Sourdough	Sardinia (IT)	Similar sensorial profile, higher acidity, higher ethanol and esters content.	[82,83,84]
	*S. bayanus × S. cerevisiae*	*De novo* hybridisation	Italy	Efficient consumption of maltotriose, appreciable level of aroma compounds.	[85]
Non- *Saccharomyces*	*Hanseniaspora guilliermondii* IST315	Grape	Portugal	Increasing eight times the content of phenylethyl acetate, associated with rose and honey aroma.	[86]
	*Hanseniaspora vineae* T02/05	Grape	Uruguay	High ester production, fruity aroma suitable for low-alcohol beer production.	[63]
	*Kazachstania servazzii*	Rye malt Sourdough	Finland	Clean flavour profile and tolerance to low-temperature conditions.	[87]
	*Lachancea fermentati* KBI 12.1	Kombucha	Ireland	Lactic acid production, lower alcohol level, fruity aroma.	[88]
	*Lachancea thermotolerans* MN477031	Grape must	Slovakia	Low lactic acid production with a minor impact on pH of the beer.	[89]
	*Mrakia gelida* DBVPG 5952	Glacial melting water	Italy	Low alcohol production and low diacetyl, and appreciable organoleptic characteristics.	[90]
	*Pichia fermentans*	Sourdough	Finland	Production of the spice/clove aroma 4-vinylguaiacol, suitable for low-alcohol wheat beers.	[87]
	*Saccharomycodes ludwigii* DBVPG 3010	Grape must	Italy	Production of low-alcohol beer, higher content of esters, and lower amount of diacetyl.	[91]
	*Torulaspora delbrueckii* DiSVA 254	Papaya leaves	Cameron	Increase of aromatic compounds, emphasised fruity/citric and fruity/esters notes.	[92,93,94]
Lactic Acid Bacteria	*L. brevis* BSO 464	Collection strain	Not reported	High flavour intensity, acidic taste, and astringency in co-fermentation.	[95,96]
	*Lactobacillus amylovorus* FST2.11	Brewing environment	Ireland	High sensitive to hops, acidification of unhopped wort until 5–6 g/L of lactic acid.	[97]
	*Pediococcus acidilactici* K10	Kimchi	Korea	Starter for malt acidification; provides bioprotection against spoilage bacteria.	[98]
	*P. acidilactici* HW01	Malt	Korea	In malt acidification, improves microbiological stability, viscosity, and filtration time.	[99,100]

**Table 4:** Cultivars, origins, and impact of adjuncts recently investigated for their brewing potential.

	Adjuncts	Regions	Impact on Beer Quality	Ref.
Fruits	Banana (cv. Prata)	Brazil	Increasing of fermentable sugars and ethanol production.	[118]
	Persimmon (cv. Rojo Brillante)	Spain	Addition of malic and citric acid, light orange colour by solubilisation of carotenoids, increase of fermentable sugars.	[117]
	Hawthorn fruit (cv. Aurea)	Not reported	Increase of antioxidant activity, polyphenols, and volatile aroma compounds.	[122]
	Cornelian cherry (cv. Podolski)	Poland	Increase of polyphenols and antioxidant activity, addition of anthocyanins and sour taste.	[119,120]
	Chestnut	Croatia	Slightly higher alcohol content, higher colour index.	[123]
	Cocoa (VP 1151)	Brazil	Increasing of wort viscosity, higher mineral, glucose and fructose content, higher ethanol production.	[124,125]
	Soursop	Brazil	Lesser variation in beer standard parameters, good acceptance in sensorial attribute.	[126]
Vegetables	Carqueja (*Baccharis trimera*)	Brazil	Addition of bittering compounds, total substitution of hop has shown no negative effects, sensorial acceptance similar to commercial beers.	[127]
	Artichoke	Brazil	Bittering effect, suitable for total hop substitution, good sensorial acceptance.	[128]
	Purple sweet potato (ST-13)	India	High content of anthocyanins and antioxidant compounds, peculiar pink colour.	[129]
	Birch-derived Xylooligosaccharides	Norway	Promoting secondary fermentation by LAB in sour beer.	[96]
	Olive leaves	Italy	Increase polyphenol content but not antioxidant activity, sour/astringent taste and herbal aroma at 10 g/L, pleasant sensory profile at 5 g/L.	[130]
	Eggplant (cv. Classic) peel extract	Romania	Increase of antioxidant activity, phenolics, and flavonoids content, reddish colour due to the release of anthocyanins.	[131]
